# Three-Dimensional Virtual Reconstruction of External Nasal Defects Based on Facial Mesh Generation Network

**DOI:** 10.3390/diagnostics14060603

**Published:** 2024-03-12

**Authors:** Qingzhao Qin, Yinglong Li, Aonan Wen, Yujia Zhu, Zixiang Gao, Shenyao Shan, Hongyu Wu, Yijiao Zhao, Yong Wang

**Affiliations:** 1 Center of Digital Dentistry/Department of Prosthodontics, Peking University School and Hospital of Stomatology & National Center for Stomatology & National Clinical Research Center for Oral Diseases & National Engineering Research Center of Oral Biomaterials and Digital Medical Devices & Beijing Key Laboratory of Digital Stomatology & NHC Key Laboratory of Digital Stomatology, Beijing 100081, China; qingzhaoq303@163.com (Q.Q.);; 2 School of Computer Science and Engineering, Beihang University & State Key Laboratory of Virtual Reality Technology and Systems, Beijing 100191, China; dragonylee@buaa.edu.cn (Y.L.);; 3 Institute of Medical Technology, Peking University Health Science Center, Beijing 100191, China

**Keywords:** external nasal defects, virtual reconstruction, deep learning, computer-aided design

## Abstract

(1) Background: In digital-technology-assisted nasal defect reconstruction methods, a crucial step involves utilizing computer-aided design to virtually reconstruct the nasal defect’s complete morphology. However, current digital methods for virtual nasal defect reconstruction have yet to achieve efficient, precise, and personalized outcomes. In this research paper, we propose a novel approach for reconstructing external nasal defects based on the Facial Mesh Generation Network (FMGen-Net), aiming to enhance the levels of automation and personalization in virtual reconstruction. (2) Methods: We collected data from 400 3D scans of faces with normal morphology and combined the structured 3D face template and the Meshmonk non-rigid registration algorithm to construct a structured 3D facial dataset for training FMGen-Net. Guided by defective facial data, the trained FMGen-Net automatically generated an intact 3D face that was similar to the defective face, and maintained a consistent spatial position. This intact 3D face served as the 3D target reference face (3D-TRF) for nasal defect reconstruction. The reconstructed nasal data were extracted from the 3D-TRF based on the defective area using reverse engineering software. The ‘3D surface deviation’ between the reconstructed nose and the original nose was calculated to evaluate the effect of 3D morphological restoration of the nasal defects. (3) Results: In the simulation experiment of 20 cases involving full nasal defect reconstruction, the ‘3D surface deviation’ between the reconstructed nasal data and the original nasal data was 1.45 ± 0.24 mm. The reconstructed nasal data, constructed from the personalized 3D-TRF, accurately reconstructed the anatomical morphology of nasal defects. (4) Conclusions: This paper proposes a novel method for the virtual reconstruction of external nasal defects based on the FMGen-Net model, achieving the automated and personalized construction of the 3D-TRF and preliminarily demonstrating promising clinical application potential.

## 1. Introduction

Nasal defects caused by oncologic resection, trauma, infection, and other diseases significantly impact patients’ facial aesthetics, causing physiological dysfunction. This not only hampers normal work and social interactions but also gives rise to severe psychological challenges [1]. Hence, enhancing the external appearance and physiological function of the defective region stands as the primary objective in nasal defect rehabilitation [2]. 

Currently, oral clinics employ two primary approaches for nasal defect rehabilitation. One is surgical reconstruction and the other involves the use of nasal prostheses. The selection of these methods depends on the size and location of the nasal defects, as well as on the patient’s wishes [3,4]. Surgical reconstruction utilizes autologous tissues, such as forehead flaps, to restore nasal defects [1,5,6]. In the conventional surgical procedure, a critical step involves the manual creation of an ideal nasal clay model by the surgeon, based on their expertise before the operation, to determine the patient’s flap shape [1]. Correspondingly, the nasal prosthesis, made of artificial materials, serves as an alternative to surgical reconstruction [2,7,8]. During the conventional prosthesis fabrication process, doctors are required to sculpt an ideal wax shape of the nasal prosthesis based on their experience and then complete the prosthesis production. Both methods for reconstructing nasal defects necessitate the manual creation of an ideal nasal solid model during preoperative design or prosthetic design, which is time-consuming, labor-intensive, and relies heavily on the surgeon’s experience. In recent years, digital technology centered around CAD/CAM has pioneered novel approaches for reconstructing nasal defects and has been successfully applied in clinical practice [8,9].

For digital-technology-assisted nasal defect reconstruction methods, a crucial step involves the utilization of a computer-aided design during the preoperative surgical design or prosthesis design phase, where the complete nasal morphology for the nasal defects is virtually reconstructed. Previous research suggests that for the virtual reconstruction of nasal defects (a type of cross-midline facial defect), an intact 3D facial model, resembling the patient’s morphology, is commonly utilized as a reference. Nasal data are then extracted from the reference facial model to restore the nasal defects [9,10,11]. In this paper, the term ‘3D target reference facial model’, abbreviated as 3D-TRF, will be used to refer to the intact 3D facial model utilized as a reference in the virtual reconstruction of nasal defects.

For the virtual reconstruction of nasal defects, the methods used in previous research to construct the 3D-TRF mainly include the following approaches. ① Database matching method: It is currently the most commonly used method for nasal-defect virtual reconstruction in oral clinical practice. This method involves establishing a facial database by collecting a large volume of 3D facial data from normal individuals. Through a customized search and matching process, the 3D facial data that closely resemble the morphology of patients with nasal defects can be selected from the database to form the 3D-TRF [10]. However, the 3D face selected from the database is not consistent with the defective face in the spatial position and has limited geometric similarity with the patient’s face before facial defects. This necessitates the further manual adjustment of the spatial position and geometric morphology of the face in the design software to achieve a good matching and overlapping effect with the patient’s defective face, which is highly dependent on the doctor’s experience and knowledge, making it laborious and time-consuming. ② The 2D-photo-to-3D-modeling method: This method relies on the patient’s 2D photo being taken before facial defects. It utilizes a 3D facial modeling algorithm, such as the Basel Face Model (BFM), to generate a complete 3D facial model. This 3D facial model is similar to the patient’s face before facial defects and can be used as the 3D-TRF [9]. However, due to the limited personalized information provided by the 2D photo, the morphological similarity between the 3D facial model generated by 3D modeling and the patient’s defective face is limited. As a result, the degree of matching and overlapping between them is low, leading to a poor degree of fit between the reconstructed nasal data and the surrounding facial tissue. ③ Deformable template method: This method utilizes a statistically shaped average face as the face template. Based on the non-rigid registration algorithm, the spatial position and geometric morphology of the face template are adjusted to match the defective facial model of the patients. The model after the deformation of the face template is used as the 3D-TRF [12]. The non-rigid registration algorithm takes the 3D face template as the reference model and the patient’s defective facial data as the target model. It achieves the non-rigid deformation of the 3D face template by solving the affine transformation matrix between the reference and target models. The non-rigidly deformed 3D face template provides nasal anatomical information for nasal defect restoration in the defective area of the patient’s face while achieving a high degree of fit between the reconstructed nasal data and the surrounding facial tissue by highly matching and overlapping with the patient’s defective face in the non-defective area. Additionally, this method achieves the adjustment of the 3D facial template through algorithm programs, reducing the dependence on the doctor’s experience to some extent and improving the automation level of 3D-TRF construction. However, its ability to reconstruct the 3D nasal morphology is limited, and 3D-TRFs constructed from the same template for different patients often have a similar nasal anatomical morphology, which cannot meet the needs of oral clinical practice for patient-personalized nasal defect reconstruction. 

In summary, the current digital construction methods for 3D-TRF in nasal-defect virtual reconstruction have yet to achieve efficient, precise, intelligent, and personalized outcomes.

In recent years, the application of deep learning models in the field of 3D shape completion has been widely studied, providing possible solutions for achieving personalized and intelligent facial-defect virtual reconstruction [13]. Currently, some scholars have conducted research on the reconstruction of cranial defects using deep learning models. For instance, Morais et al. [14] designed a volumetric convolutional denoising autoencoder based on convolutional neural networks. Trained on voxel data obtained from head MRI scans, this model learns volume representations to effectively and automatically reconstruct cranial defects. Xu et al. [15] constructed a deep learning model, RecGAN, based on a conditional generative adversarial network (cGAN) to achieve 3D shape completion by repairing defective CT images layer by layer. RecGAN demonstrates the intelligent reconstruction of midfacial bone defects, adapting well to the unique conditions of different patients. Research on the reconstruction of cranial hard tissue defects using deep learning models has shown promising results in achieving precise, intelligent, and personalized cranial defect reconstruction [14,15,16,17,18,19]. However, there is currently no reported research on the application of deep learning models for facial soft-tissue defect reconstruction. 

Therefore, for the virtual reconstruction of nasal defects (cross-midline facial defects), we propose a deep learning model, the Graph Convolution-Based Facial Mesh Generation Network (FMGen-Net), to construct the 3D-TRF. We also quantitatively evaluated the 3D-TRF construction effectiveness of the FMGen-Net model based on the metrics of ‘morphological similarity’ and ‘edge fitness’, aiming to provide a reference for the clinical application of the algorithm.

Our contributions are as follows:

- Introducing a new method for the virtual reconstruction of full nasal defects.
- Designing a deep learning model, FMGen-Net, based on three-dimensional surface data types.
- Constructing a structured 3D facial dataset for training and testing the FMGen-Net model.
- Improving the automation and personalization of the virtual reconstruction of full nasal defects based on deep learning models.

## 2. Materials and Methods

**Figure 1:**
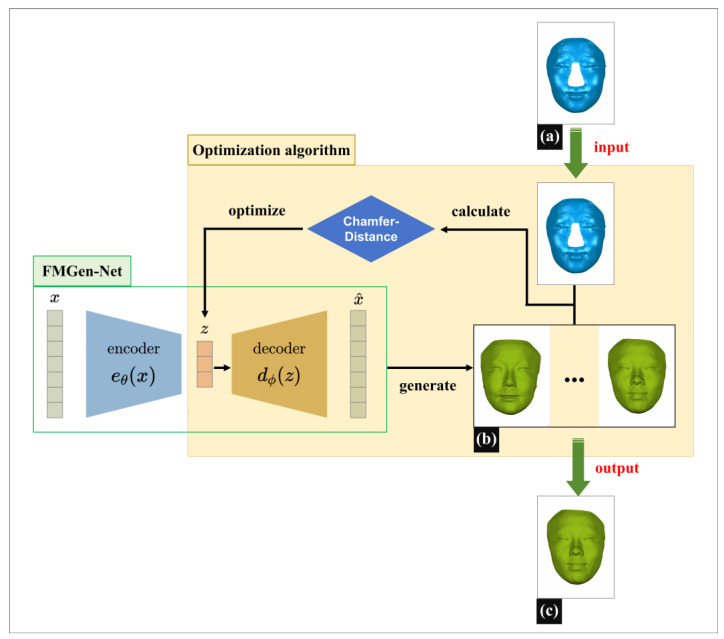
The principle of constructing 3D-TRFs based on the FMGen-Net model. (**a**) The defective face of a patient (blue face). (**b**) The faces randomly generated by the trained decoder module, which takes a latent space vector as the input (green face). (**c**) 3D-TRF, the face eventually constructed through iterating the faces randomly generated by the decoder, which matches the patient’s defective face.

This study proposed an automatic construction algorithm for 3D-TRFs using the FMGen-Net deep learning model as its core, characterized by detaching the process of learning to generate 3D faces from the task of constructing 3D-TRFs, as illustrated in Figure 1. 

The FMGen-Net model is an autoencoder consisting of two modules: an encoder and a decoder. It is an unsupervised learning model. By inputting a normal person’s 3D facial data (x) itself as supervision, the neural network was trained to generate a 3D face $(\tilde{x}$) similar to x to learn the 3D face generation process. After FMGen-Net training was completed, the network parameters of the encoder and decoder were fixed, the encoder module was discarded, and the decoder module was used as the 3D face generator to construct the 3D-TRF for the patient. The decoder module took a latent space vector as the input to generate a 3D face that conformed to facial anatomical morphology. However, the generated face was entirely random and lacked personalized anatomical morphology matching the patient’s defective face. In this study, an optimization algorithm was employed to construct personalized 3D-TRFs for patients. By iterating the faces randomly generated by the decoder module, the difference between the generated face and the patient’s defective face was continuously reduced, eventually generating a complete face that matched the patient’s defective face. In the following sections, we describe, in more detail, the ingredients of our process, the FMGen-Net training, and the optimization algorithm for constructing the 3D-TRFs. 

### 2.1. Constructing a Structured 3D Facial Dataset for Model Training and Testing

We recruited 400 adult patients receiving treatment at Peking University School and the Hospital of Stomatology to build a 3D facial dataset. Inclusion criteria were as follows: (1) normal facial morphology without significant deformities or noticeable asymmetry and (2) absence of soft tissue defects and severe facial trauma. Exclusion criteria included sensitivity to white light and unwillingness to undergo 3D facial optical scanning. The study received approval from the Bioethics Committee of Peking University Hospital of Stomatology (PKUSSIRB-202385001). The patients were informed about the research purpose and signed informed consent forms.

**Figure 2:**
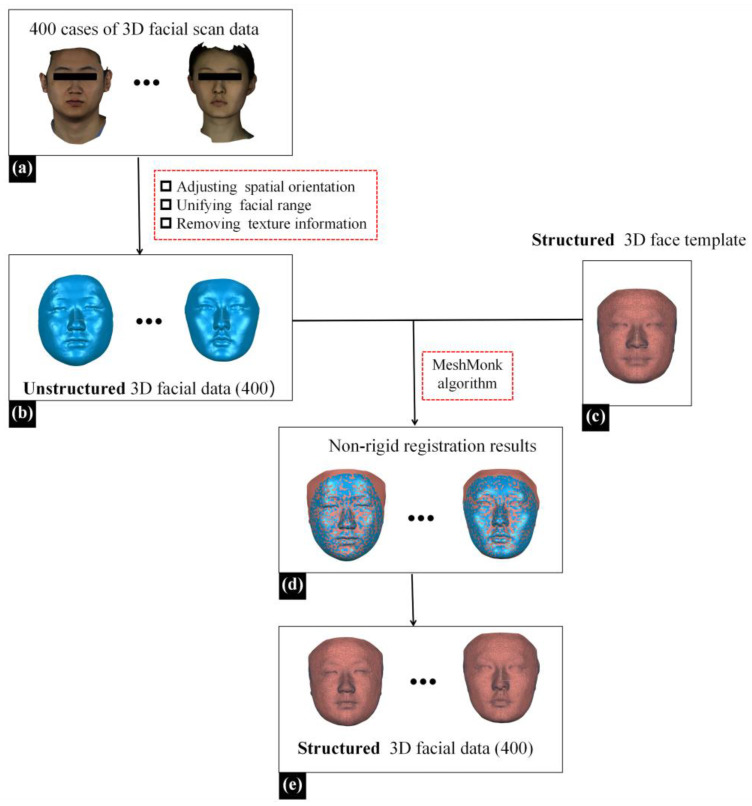
The process of constructing a structured 3D facial dataset. (**a**) The 3D scanned facial data of 400 patients. (**b**) The 3D facial data after preprocessing, which were the ‘unstructured’ triangular mesh data (blue face). (**c**) The 3D face template, which comprised the ‘structured’ triangular mesh data (reddish-brown face). (**d**) The results of non-rigid registration of the structured face template to unstructured patient facial data (blue and reddish-brown interlacing face). (**e**) The resulting structured 3D facial data, which comprised the deformed template (reddish-brown face).

Three-dimensional facial data of 400 patients were collected using a 3D facial scanning device (Face Scan, 3D-Shape Crop, Erlangen, Germany). Face Scan is based on white light-grating scanning, operated with a scanning angle of 270°–320°, a scanning speed of 0.8 s, and a scanning accuracy of 0.2 mm. The 3D facial data obtained through scanning were unstructured, disordered mesh data, with approximately 10,000 data points and 20,000 triangular meshes. During the 3D facial scanning using Face Scan, patients were required to sit 135 cm in front of the instrument, maintain a natural head position, gaze straight ahead with a relaxed facial expression, and ensure there was no hair obstruction on the face. The scanned data, as illustrated in Figure 2a, were saved in .obj format.

Next, the Geomagic Wrap 2021 software (3D Systems, Rock Hill, SC, USA), a reverse engineering software, was utilized to preprocess the above facial data [12]. (1) A senior physician from the Department of Oral Prosthodontics manually adjusted the spatial orientation of the data, aligning the YZ plane parallel to the mid-sagittal plane and the XZ plane parallel to the Frankfurt plane (FH plane) to achieve a natural head position. (2) Redundant data were deleted, unifying the facial range from the hairline up to the subchin, and extending from the left to the right of the tragus on both sides. (3) Texture data were deleted, preserving only geometric information, as illustrated in Figure 2b.

Due to the FMGen-Net model proposed in this study being designed based on a graph convolutional neural network, the model training required utilizing facial data in the ‘structured’ triangular mesh data format to learn the representation of 3D facial shapes [20,21]. This required unifying the topology of the 3D facial data in the dataset, ensuring that the numbers of points and meshes for all 3D facial data were the same, and that the same numbered points corresponded to the same anatomical position within the 3D facial data. Therefore, we processed the unstructured facial data obtained from the above 3D face scanning to construct a structured 3D facial dataset. For this purpose, we employed a structured 3D face template and the Meshmonk non-rigid registration algorithm [22,23,24]. By non-rigidly registering the structured 3D face template to the unstructured patient facial data, we constructed the structured patient facial data with the same topological structure as the structured 3D face template, as illustrated in Figure 2e. The structured facial template used in this study was the average facial data constructed by our research team based on 30 normal 3D facial data in the previous study, comprising 9856 points and 19,534 triangular meshes [24]. Based on the above methods, we constructed a structured 3D facial dataset of 400 cases.

### 2.2. Training and Testing the FMGen-Net Model

The structured 3D facial dataset constructed in Section 2.1, consisting of 400 cases, was divided into training, validation, and testing sets in a ratio of 17:2:1 for training and testing the FMGen-Net model. The training and validation sets were used to train the model to learn the process of generating 3D faces while the testing set was used to evaluate the model’s ability to generate 3D faces.

**Figure 3:**
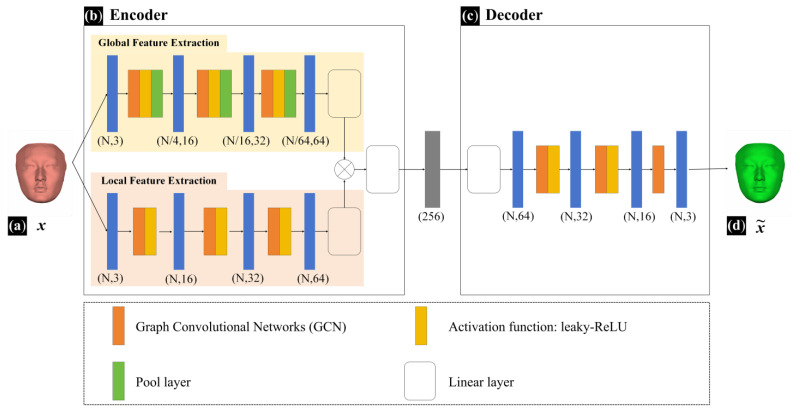
The network structure of the FMGen-Net model. (**a**) *x* represents the input intact structured facial data (reddish-brown face). (**b**) The encoder module extracts feature from intact facial data, ultimately obtaining a 256-dimensional latent space vector. (**c**) The decoder module takes a latent space vector as input to generate a new intact 3D face. (**d**) $\tilde{x}$ represents the facial data generated by the decoder module (bright green face).

The FMGen-Net model established in this study is an autoencoder comprising two modules: an encoder and a decoder, as illustrated in Figure 3. 

The encoder module has approximately 300,000 parameters. It processes mesh data using Graph Convolutional Networks (GCNs) to extract geometric features from structured facial data [21]. Feature extraction within the encoder module encompasses two branches: global feature extraction and local feature extraction. Global feature extraction captures the overall shape information of the 3D face while local feature extraction focuses on detailed local shape information. The global-feature-extraction branch consists of 10 network layers, comprising 3 repeated graph convolutional layers, activation layers, pooling layers, and 1 linear layer. The local feature extraction branch consists of 6 network layers, comprising 3 repeated graph convolutional layers, activation layers, and 1 linear layer. Inputting intact structured facial data (*x*) into the encoder module, it extracts both global and local features, performs feature fusion, and ultimately obtains a 256-dimensional latent space vector. The decoder module also has approximately 300,000 parameters and consists of 5 network layers, comprising 2 repeated graph convolutional layers, activation layers, and 1 graph convolutional layer. It takes a latent space vector as the input to generate a new intact 3D face ($\tilde{x}$). The activation function for both the decoder and encoder modules of FMGen-Net was set to leaky-ReLU.

During the training of the FMGen-Net model, we optimized network parameters by minimizing the reconstruction loss, that is, by training FMGen-Net to make the generated $\tilde{x}$ and the input *x* as similar as possible. Since the generated $\tilde{x}$ and the input *x* share the same topological structure, we employed mean squared error (MSE) as the reconstruction loss function, as shown in Formula (1). MSE evaluates the reconstruction loss between generated and input faces by computing the sum of squared distances between corresponding points of x and $\tilde{x}$.

(1) $$L_{\mathrm{recon}}=MSE\left(x,\tilde{x}\right)$$

Here, x represents the input intact 3D facial data and $\tilde{x}$ represents the generated new 3D facial data.

In addition, we incorporated a regularization loss function to impose spherical regularization constraints on the latent space of FMGen-Net. This helped the latent space vectors within a hypersphere distribution, thereby facilitating faster convergence during the optimization process for generating facial data by the decoder, as shown in Formula (2).

(2) $$L_{\mathrm{reg}}={\left(\left|z\right|-1\right)}^{2}$$

Here, z represents a vector in the latent space.

In summary, the loss function configuration during the training of FMGen-Net is represented by Formula (3).

(3) $$L=L_{recon}+{\lambda L}_{reg}$$

Here λ represents the weights of $L_{reg}$, serving as the tuning parameters during network training.

After constructing the network model, we compiled it by manually setting the hyperparameters. During the model compilation, we used empirical values for the hyperparameters. For example, the weight of the regularization loss function could be set to 0.1, 0.01, or 0.001, and the initial learning rate could be set to 0.01, 0.001, or 0.0001. By trying different combinations of hyperparameter values and observing the changes in the loss curves on the training and validation sets, we finally determined the optimal hyperparameter values. The hyperparameter finally chosen was as follows: λ=0.01, and the optimizer was Adam with an initial learning rate of 0.001. 

FMGen-Net was implemented based on Python 3.10 and PyTorch 2.0, compiled on the Ubuntu 22.04 operating system running on an AMD EPYC 7B12 CPU equipped with an NVIDIA RTX 4090 GPU and with a total running memory of 128 GB.

After training the FMGen-Net model, the model’s ability to generate 3D faces was evaluated using the testing set. The mean squared error (MSE) was used as the evaluation metric to quantitatively assess the difference between the 3D faces generated by the FMGen-Net model and the original faces in the test set, assessing the model’s performance.

### 2.3. Applying an Optimization Algorithm to Construct 3D-TRFs for Patients with Nasal Defects

**Figure 4:**
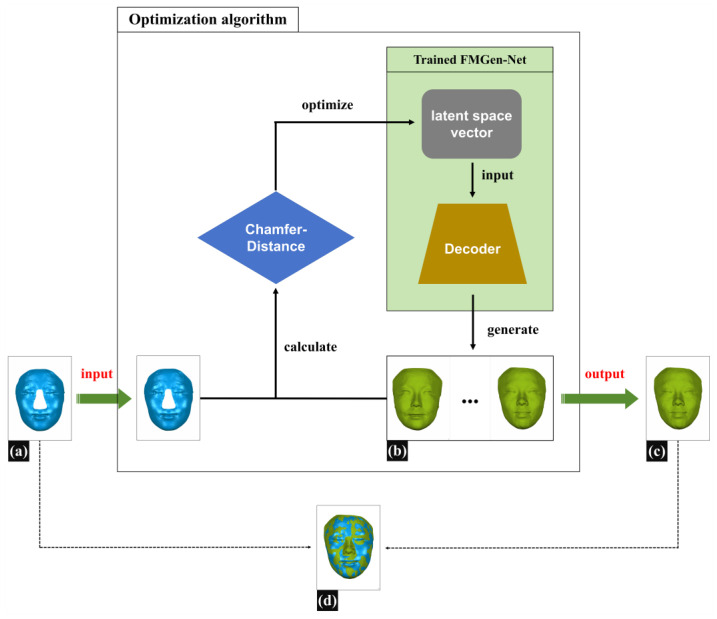
The schematic diagram of constructing 3D-TRFs using optimization algorithms. (**a**) Face_Defect, the defective face of a patient (blue face). (**b**) Face_Generator, the faces randomly generated by the trained decoder module, which takes a latent space vector as the input (green face). (**c**) 3D-TRF, the face eventually constructed through iterating the faces randomly generated by the decoder, which matches the patient’s defective face. (**d**) The overlap effect of 3D-TRF with the patient’s defective face (blue and green interlacing face).

Figure 4 illustrates the schematic diagram of constructing 3D-TRFs using optimization algorithms. During the virtual reconstruction of nasal defects, doctors typically need to determine the boundary of the defect range on the patient’s 3D facial scan data, delete the data within the defect range, and then restore the defect by constructing a 3D-TRF. In this study, we employed a simulated defect creation approach to construct defective facial data that resemble clinical scenarios: on normal 3D facial scan data, we determined the boundary of the nasal defect range, deleted normal nasal data within the defect range, and created defective facial data named Face_Defect. The detailed construction method for defective facial data is described in Section 2.4. This section will introduce the method for constructing personalized 3D-TRFs for patients based on the FMGen-Net model.

The task of constructing a 3D-TRF for patients with nasal defects in this study was regarded as a facial generation task. Once FMGen-Net has been trained, the network parameters of the encoder–decoder module are fixed, and the encoder is essentially tossed away, while the decoder acts as a generator to generate intact face. The decoder module takes a latent space vector as the input to generate a 3D face, referred to as Face_Generator. However, the generated face is entirely random and lacks personalized anatomical morphology matching the patient’s defective face, and thus cannot be used directly as a 3D-TRF to reconstruct nasal defects. In this study, an optimization algorithm was employed to construct personalized 3D-TRFs for patients.

Assuming the defective facial data (Face_Defect) are denoted as $S_{1}$ and the generated face (Face_Generator) as $S_{2}$, $S_{2}=Decoder(z)$, we defined the unidirectional chamfer distance (CD) as $\mathrm{CD}\left(S_{1},S_{2}\right)$, according to Formula (4).

(4) $$\mathrm{CD}\left(S_{1},S_{2}\right)=\frac{1}{\left|S_{1}\right|}\sum_{x\in S_{1}}\underset{y\in S_{2}}{min}|x-y{|}_{2}^{2}$$

Here, $S_{1}$ represents the input defective face (Face_Defect), $S_{2}$ is the generated intact face (Face_Generator), x is a vertex in $S_{1}$, and y is a temporary variable representing the nearest corresponding point in $S_{2}$ to x.

Calculating the difference between the generated face $S_{2}$ and the defective face $S_{1}$ iteratively solves for the values of the latent space vector z and ensures that the z vector conforms to a hypersphere distribution. The goal of the iterative solution was to minimize $\mathrm{CD}\left(S_{1},S_{2}\right)$, as shown in Formula (5). Ultimately, an intact face was generated that was most similar to the remaining anatomical region of the patient’s defective face, and this served as the 3D-TRF.

(5) $$R^{*},T^{*},z^{*}=\underset{R,T,z}{argmin}\;\mathrm{CD}\left(S_{1},R*S_{2}+T\right)+\lambda L_{\mathrm{reg}}$$

Here, $L_{\mathrm{reg}}$ is the regularization loss function, and R and T are the rotation matrix and translation vector, respectively. z is a latent space vector. Applying spatial rotation and translation to the generated Face_Generator helps correctly find the corresponding relationship with Face_Defect’s nearest points. This adjustment ensures that Face_Generator occupies the same spatial position as Face_Defect in the world coordinate system.

Based on the prior facial shape knowledge learned by FMGen-Net, the intact facial data finally generated by the decoder exhibited personalized 3D anatomical morphology. It closely matched and overlapped with the patient’s defective face in non-defective regions. Simultaneously, it provided personalized 3D nasal shape information in the defective area. Therefore, it could be used as the 3D-TRF, offering valuable references for nasal-defect virtual reconstruction, as illustrated in Figure 4d.

### 2.4. Evaluating the Nasal Defects Reconstruction Effectiveness of 3D-TRFs Constructed by FMGen-Net

This study evaluated the effectiveness of personalized 3D-TRFs constructed by FMGen-Net in nasal defect reconstruction through a simulated experiment of full nasal defects. The aim was to preliminarily validate the feasibility of the FMGen-Net model in clinical applications. For this purpose, we newly collected 20 cases of normal human 3D facial scanning data for constructing the simulated defective facial data. We then evaluated the virtual reconstruction effects of nasal defects using clinical indicators of interest to doctors.

First, a set of facial data with full nasal defects were constructed based on 20 normal 3D facial scans. Then, the 3D-TRF was generated by the FMGen-Net model using the optimization algorithm, and the full nasal defects was reconstructed based on the 3D-TRF. To objectively assess the reconstructive efficacy of 3D-TRF for full nasal defects, we quantitatively evaluated the reconstruction outcomes of nasal defects using two metrics: ‘morphological similarity’ and ‘edge fitness’. The details are as follows.

#### 2.4.1. Constructing Facial Data with Nasal Defects

In this study, facial data with full nasal defects were constructed using 20 newly collected normal 3D facial scanning data, ensuring that these facial data were not learned by FMGen-Net. These 20 facial scanning data underwent the preprocessing procedures described in Section 2.1, including adjusting the spatial pose and unifying the facial range. In the reverse engineering software (Geomagic Wrap 2021), the range of full nasal defects was determined on the normal face using the ‘Create Boundary From Spline’ feature, encompassing areas above the nasal root, bilaterally inside the inner canthus, and below the nasal columella point. Subsequently, the ‘New Object from Selection’ feature was utilized to extract facial data outside the full nasal range, denoted as the defective facial data (Face_Defect). Additionally, nasal data within the defect range were extracted and named Nose_Original, serving as the ground truth for comparison with reconstructed nasal data.

#### 2.4.2. Virtual Reconstruction of Full Nasal Defect Based on Personalized 3D-TRF

**Figure 5:**
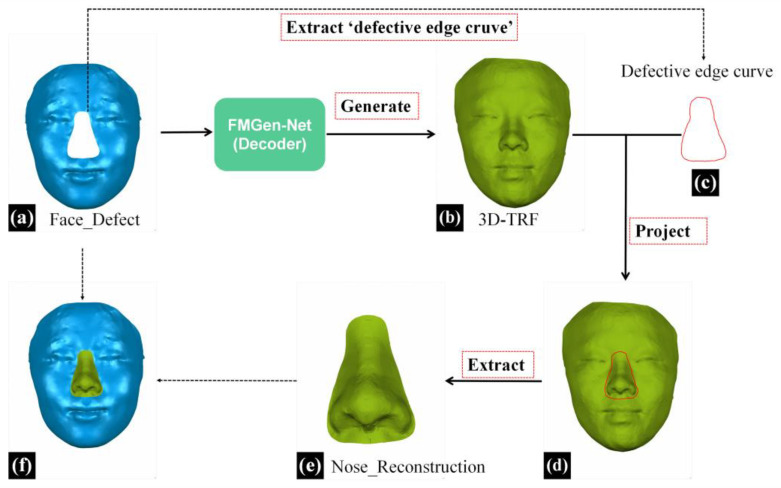
The reconstruction process of nasal defects based on a personalized 3D-TRF. (**a**) The defective face of a patient, Face_Defect (blue face). (**b**) The face eventually generated by the decoder module, serving as 3D-TRF (green face). (**c**) The curve extracted from the defective face, named ‘Defective edge curve’, represents the range of full nasal defects. (**d**) The result of ‘Defective edge curve’ being projected onto the 3D-TRF. (**e**) Nose_Reconstruction, the nasal data extracted from 3D-TRF (green nose). (**f**) The reconstructed results for patients with full nasal defects (blue face with green nose).

This study employed the 3D morphological reconstruction method for external nasal defects, previously investigated by our research group, to extract nasal data from the 3D-TRF for reconstructing the nasal defects [12]. An overview of the entire virtual reconstruction process for full nasal defects is presented in Figure 5.

In the reverse engineering software (Geomagic Wrap, 2021), the process began with the initial extraction of the defective edge curve from the facial data with full nasal defects (Face_Defect). Subsequently, this curve was projected onto the 3D-TRF and transformed into a boundary curve. Further, the ‘New Object from Selection’ feature was utilized to extract nasal data within the defective range on 3D-TRF, resulting in the obtained reconstructed nasal data, which were named Nose_Reconstruction, as illustrated in Figure 5e.

#### 2.4.3. Evaluating the Reconstruction Effectiveness with the ‘Morphological Similarity’ Metric

This paper defines the ‘morphological similarity’ metric as the three-dimensional resemblance between reconstructed nasal data and original nasal data, reflecting the anatomical morphology restoration effectiveness of full nasal defect reconstruction.

**Figure 6:**
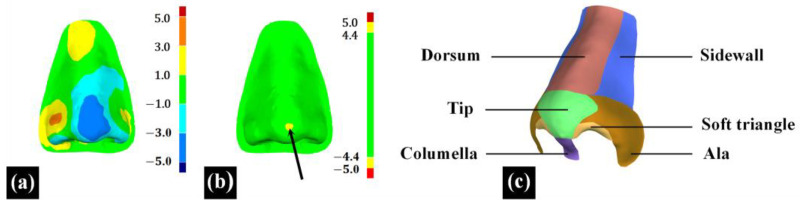
The evaluation of the ‘morphological similarity’ metric. (**a**) The color map of ‘3D compare’ analysis. (**b**) The nasal area observed with ‘max surface deviation’, as indicated by the black arrow. (**c**) Nasal subunits.

In the 3D measurement and analysis software Geomagic Control X 2023 (3D System, Rock Hill, SC, USA), the ‘3D Compare’ feature was employed to calculate the root mean square deviation (RMSD) between two point cloud data, termed ‘3D surface deviation’. Additionally, the maximum deviation (Max-Deviation) between the two point cloud data was calculated and termed as the ‘max surface deviation’, with the location of the max surface deviation recorded in nasal subunits, as indicated by the black arrow in Figure 6b. The ‘3D surface deviation’ represents the overall difference between the two point cloud data, as illustrated in Figure 6a. A smaller ‘3D surface deviation’ indicates a greater similarity in 3D morphology between the reconstructed nasal data and the original nasal data. The ‘max surface deviation’ represents the maximum distance between corresponding points within the two point cloud data, indicating the extent of the maximum 3D morphological difference between the reconstructed nasal data and the original nasal data. Based on the principle of subunits in nasal defect reconstruction [25,26], this study divided the full nose into nine subunits, including the nasal dorsum, nasal sidewalls, nasal tip, nasal columella, soft triangles, and nasal alae, to assess the distribution of the ‘max surface deviation’ of 20 reconstructed noses across different subunits, as shown in Figure 6c.

#### 2.4.4. Evaluating the Reconstruction Effectiveness Using the ‘Edge Fitness’ Metric

**Figure 7:**
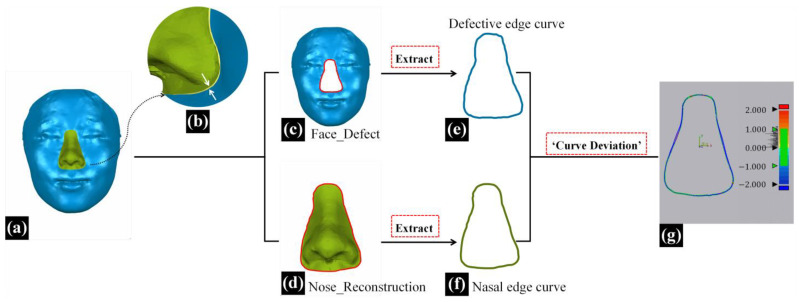
The evaluation process of the ‘edge fitness’ metric. (**a**) The reconstructed results for patients with full nasal defects (blue face with green nose). (**b**) The reconstructed nasal data (green nose) do not seamlessly connect with the surrounding soft tissues of the defective face (blue face) at the defect boundary, as indicated by the white arrows. (**c**) Face_Defect, the defective face of a patient (blue face). (**d**) Nose_Reconstruction, the nasal data extracted from 3D-TRF (green nose). (**e**) The ‘Defective edge curve’ extracted from the defective facial data (blue curve). (**f**) The ‘Nasal edge curve’ extracted from the reconstructed nasal data (green curve). (**g**) The color map of ‘Curve Deviation’ analysis.

In addition to morphological similarity evaluation, the degree of fitness between the reconstructed nasal data and the surrounding soft tissues of the defective face can also impact the effectiveness of nasal defect reconstruction [11]. In this study, as shown in Figure 4d, the 3D-TRF and the defective face did not completely overlap in the non-defective area, and the reconstructed nasal data (Nose_Reconstruction) extracted from the 3D-TRF did not seamlessly connect with the surrounding soft tissues of the defective face at the defect boundary, as indicated by the white arrows in Figure 7b. In this study, the ‘edge fitness’ metric was defined as the degree of fitness between the reconstructed nasal data and the surrounding soft tissues of the defective face [12]. An overview of the entire process for evaluating ‘edge fitness’ is presented in Figure 7. 

In the three-dimensional measurement and analysis software Geomagic Control X 2023, the ‘defective edge curve’ on the defective face and the ‘nasal edge curve’ on the reconstructed nose were extracted separately using the ‘Boundary’ feature. Subsequently, the ‘Curve Deviation’ function was employed to calculate the root mean square deviation (RMSD) between the corresponding point pairs of the two curves, termed the ‘curve deviation’. Additionally, the maximum deviation (Max-Deviation) among these corresponding point pairs of the two curves was calculated and termed as the ‘max curve deviation’. The ‘curve deviation’ represents the overall difference between the two curves. A smaller ‘curve deviation’ indicates a better fit between the reconstructed nose and the surrounding soft tissues of the defective face, signifying better effective nasal defect reconstruction. The ‘max curve deviation’ represents the maximum distance between the two curves, indicating the extent to which the reconstructed nasal data least fit with the surrounding soft tissue.

#### 2.4.5. Statistical Analysis

Using SPSS 26.0 software (IBM, Armonk, NY, USA), descriptive statistical analysis was conducted for the evaluation of the virtual reconstruction effectiveness of full nasal defects in the aforementioned 20 cases. Normality tests were performed separately for the evaluation results of the ‘morphological similarity’ metric, namely ‘3D surface deviation’ and ‘max surface deviation’, and of the ‘edge fitness’ metric, namely ‘curve deviation’ and ‘max curve deviation’. If the variables conformed to a normal distribution, the central tendency and dispersion trends were represented using the mean ± standard deviation. In cases where normal distribution assumptions were not met, representation was denoted using the median (interquartile range).

## 3. Results

### 3.1. The Results of FMGen-Net Model Training and Testing

In the training of the FMGen-Net model, the reconstruction losses (MSEs) of the training set and validation set were 0.295 and 0.575, respectively. This indicates that through model training, FMGen-Net had learned prior knowledge of 3D facial anatomy and could generate 3D faces that conform to facial anatomical morphology.

When evaluating the model performance using the testing set, the average MSE was 0.749. This suggests that FMGen-Net has a good capability to generate 3D faces and can generate 3D faces that are morphologically similar to those not encountered during training.

Given the good performance of FMGen-Net on the testing set, its decoder module can be used as a 3D face generator. By applying optimization algorithms, it can be used to construct 3D-TRFs for patients with full nasal defects.

### 3.2. The Results of Simulated Experiment of Full Nasal Defects

**Figure 8:**
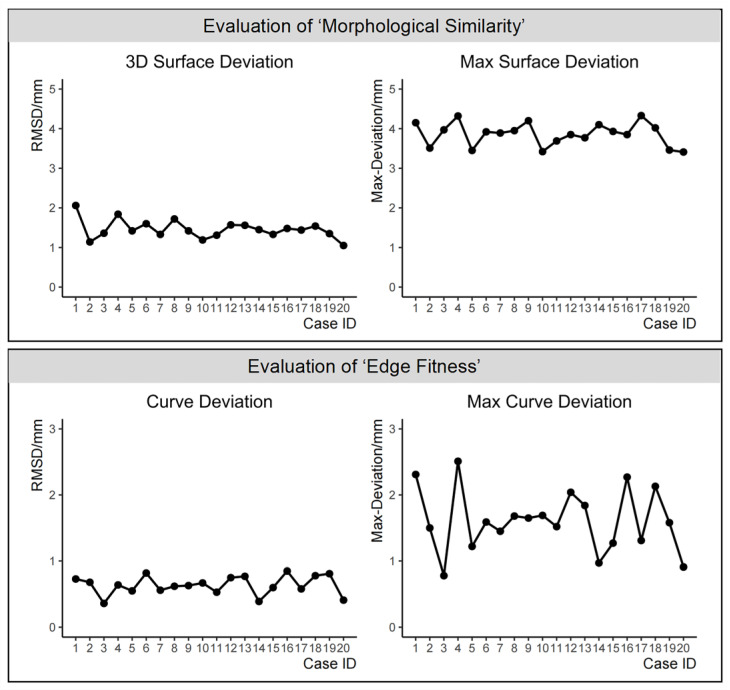
The evaluation results of the ‘morphological similarity’ metric and the ‘edge fitness’ metric in 20 cases of a full nasal defect reconstruction.

For the simulation experiment of 20 cases of a full nasal defect reconstruction, Figure 8 separately shows the evaluation results of the ‘morphological similarity’ metric, namely ‘3D surface deviation’ and ‘max surface deviation’, and the ‘edge fitness’ metric, namely ‘curve deviation’ and ‘max curve deviation’.

**Figure 9:**
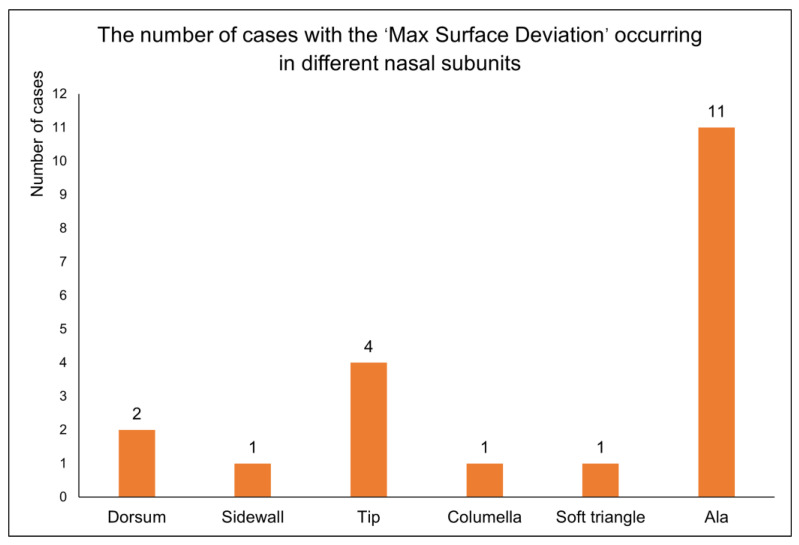
The numbers of cases with the ‘max surface deviation’ occurring in different nasal subunits.

The descriptive statistical analysis of the evaluation results for the virtual reconstruction of 20 cases with full nasal defects is as follows: in the evaluation of ‘morphological similarity’ between reconstructed nasal data and original nasal data, both ‘3D surface deviation’ and ‘max surface deviation’ followed a normal distribution. The 3D surface deviation was 1.45 ± 0.24 mm and the ‘max surface deviation’ was 3.86 ± 0.29 mm. The number of cases with the ‘max surface deviation’ occurring in different nasal subunits is illustrated in Figure 9. Specifically, in eleven cases, the ‘max surface deviation’ was observed in the nasal alae, and in four cases in the nasal tip; in fewer cases, it was noted in the nasal dorsum, nasal sidewalls, soft triangles, and nasal columella. 

Regarding the evaluation of ‘edge fitness’ between reconstructed nasal data and surrounding soft tissues, both ‘curve deviation’ and ‘max curve deviation’ followed a normal distribution. The ‘curve deviation’ was 0.64 ± 0.14 mm and the ‘max curve deviation’ was 1.61 ± 0.47 mm.

**Figure 10:**
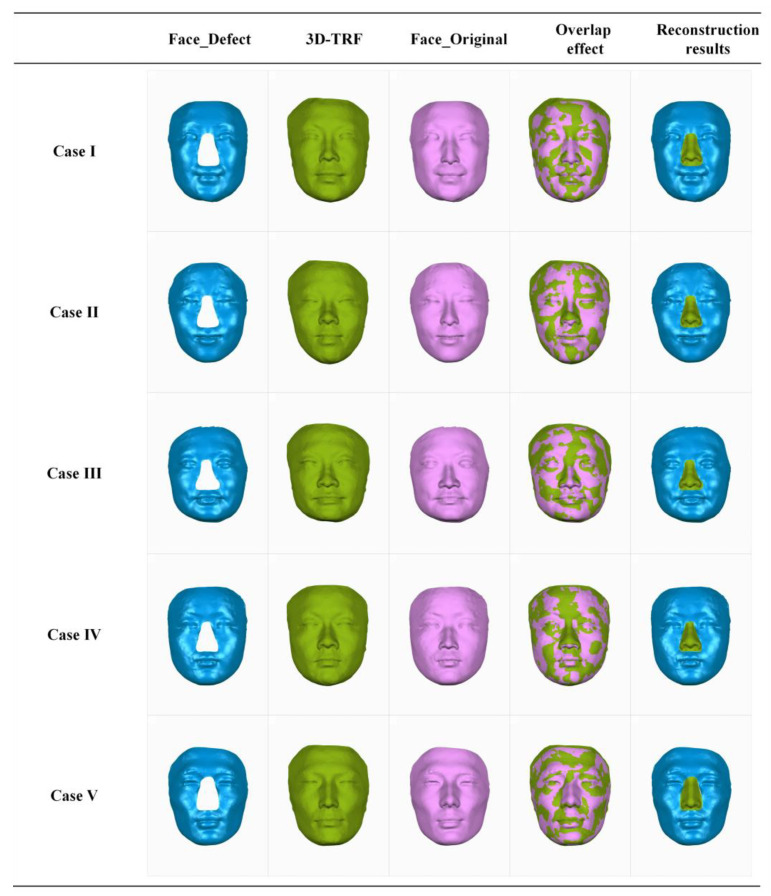
The virtual reconstruction results of full nasal defects in some cases. Blue face: Face_Defect, the defective face constructed from a normal face. Green face: The face eventually generated by the decoder module, serving as 3D-TRF. Pink face: Normal original facial data used to simulate the construction of a defective face. Pink and green interlacing face: The overlapping effect between the 3D-TRF and the Face_Original. Blue face with green nose: Reconstruction results for Face_Defect.

Figure 10 showcases the virtual reconstruction results of full nasal defects for a subset of cases from the 20 simulated defect cases. From a frontal view, it illustrates the simulated full nasal-defect face, the 3D-TRF constructed based on the FMGen-Net model, the original intact face (named Face_Original), the overlap effect between the 3D-TRF and the original intact face, and the reconstruction results of full nasal defects based on the 3D-TRF. It is evident that the 3D-TRF generated by the FMGen-Net model closely resembles the original facial morphology, achieving a satisfactory reconstruction of nasal defects.

## 4. Discussion

### 4.1. The 3D Virtual Reconstruction of Nasal Defects Based on the FMGen-Net Model Demonstrates Promising Clinical Application Potential

In the virtual reconstruction of nasal defects, efficiently and accurately reconstructing the complete 3D morphology of nasal defects is a crucial issue. Previous studies using deep learning algorithms for the reconstruction of cranial-bone hard tissue defects have shown that the application of deep learning models can achieve personalized cranial defect reconstruction results and improve the automation level of the cranial defect reconstruction process, thereby reducing the dependence on physician experience. Given the current lack of reports on the application of deep learning algorithms to achieve the reconstruction of facial soft tissue defects, this study innovatively introduced deep learning algorithms into the virtual reconstruction of nasal defects. By training the artificial intelligence model FMGen-Net and combining it with an optimization algorithm, personalized 3D-TRFs are automatically constructed for patients with nasal defects, preliminarily achieving the efficient and accurate virtual reconstruction of nasal defects.

At oral clinics, the database matching method is currently most commonly used in the virtual reconstruction of nasal defects. Zeng et al. [10] proposed an automatic retrieval scheme based on three-dimensional image registration technology, which automatically selects the most similar face to the patient from a three-dimensional facial database as the 3D target reference data. This approach improves the retrieval efficiency of the database matching method. Reitemeier et al. [27], on the other hand, developed a custom software to fit the selected nasal data from the database onto the patient’s three-dimensional facial scan data. They achieved the rapid positioning and morphological adjustment of the virtual nose by using features such as “Zoom” and “Margin adaptation”. Sun et al. [11] proposed a non-rigid registration algorithm to replace manual adjustments of detected faces in the database. They used the detected face from the database as a template and deformed it to match the defective face, automatically adjusting the spatial position and geometric morphology of the detected face using a non-rigid registration algorithm. This approach further improves the efficiency of nasal-defect virtual reconstruction using the database matching method. The studies mentioned above fully explored the clinical application potential of the database matching method. However, their method relies on the establishment of databases, and due to differences in data acquisition methods and research center practices, databases are often not universally applicable across different hospitals and lack generalizability. In this regard, Wen et al. [12] proposed the deformable template method. They collected three-dimensional facial data from a certain number of normal individuals and calculated an average face template that has anatomical representativeness. By using this face template and applying non-rigid registration algorithms to adjust its spatial position and geometric morphology to match the defective face, they eliminated the need for database reliance. This method improves the algorithm’s universality across different hospitals. Additionally, benefiting from the strong deformation capability of non-rigid registration algorithms, the deformed face template fits well with the defective face, and the reconstructed nasal data has good fitness with the surrounding soft tissue edges. However, its limitation lies in the fact that although the average face template contains three-dimensional morphological information with anatomical representativeness, it lacks personalized information that matches the patient. As a result, the deformed face template typically has similar nasal anatomical information, which fails to meet the needs of oral clinical practice for personalized nasal reconstruction. Compared with the two methods mentioned above, the virtual reconstruction method for nasal defects introduced in this study, based on deep learning models, no longer requires a reliance on database use in clinical applications. This improves the algorithm’s universality across different hospitals. Thanks to the learning ability of the AI model, a three-dimensional target reference face with personalized anatomical morphological information can be constructed, and this will be fully suitable for the specific situations of different patients.

In previous studies, two other novel attempts to improve the automation and personalization of nasal defect reconstruction were the 2D-photo-to-3D-modeling method and the statistical-shape-model method. Currently, there are few reports on these two methods. The 2D-photo-to-3D-modeling method requires the use of normal 2D photos of the patient before facial defects, and based on the limited information provided by 2D photos, the 3D model generated may have limited personalized anatomical morphological information. One promising direction for the future advancement of this method is to combine deep learning algorithms with 3D modeling algorithms to enhance the facial generation capabilities of 3D modeling algorithms. The statistical-shape-model method obtains the main anatomical morphological change information of normal faces through statistical analysis. When inputting defective facial data, it utilizes the remaining anatomical morphological information of the defective face to calculate and generate a complete face with personalized anatomical morphology [28]. In the study by Swanepoel et al. [23], which employed a 3D facial statistical shape model to predict defective facial tissue, the root mean square value used for assessing the 3D surface deviation between the predicted nose and the original nose was reported as 2.10 ± 0.59 mm. In our study, a morphology similarity assessment between the reconstructed nasal data and the original nasal data was conducted in 20 cases of simulated full nasal defects. The evaluation showed a three-dimensional surface deviation of 1.45 ± 0.24 mm. Compared to the statistical shape model method, we proposed a better anatomical morphology restoration effect for nasal defects. This may have been due to the limited main anatomical morphological change information obtained by the statistical shape model, which makes it difficult to fully adapt it to the specific situations of different patients [29]. 

In summary, the nasal-defect virtual reconstruction method proposed in this study, which involves training the deep learning model FMGen-Net and combining it with an optimization algorithm, can automatically construct 3D-TRFs with personalized anatomical morphological information for patients. This method has improved the level of automation in nasal-defect virtual reconstruction to a certain extent and reduced the dependence on physician experience. Compared to various algorithms proposed in previous studies, it has better advantages in clinical application.

### 4.2. Limitations and Prospects

In the method used in this study, due to the fact that the FMGen-Net model does not directly complete a defective face but generates an intact face matching the remaining anatomical information of the defective face, the generated 3D-TRF does not completely overlap with the defective face. The reconstructed nasal data constructed based on 3D-TRFs do not seamlessly connect with the surrounding soft tissues of the defective face. By defining the ‘edge fitness’ metric, we quantitatively assessed the degree of fitness between the reconstructed nasal data and the surrounding soft tissues. The curve deviation was measured at 0.64 ± 0.14 mm. In previous research by Wen et al., a deformable template method was employed for the virtual reconstruction of full nasal defects using the model deformed from the average face template as the 3D-TRF. In the evaluation of the ‘edge fitness’ metric, the curve deviation was measured at 0.37 ± 0.09 mm [12]. This indicates that compared to the deformable template method, there is potential for improvement in the alignment and overlap between the personalized 3D-TRF and the defective face.

The deformable template method benefits from the deformable matching capability of non-rigid registration algorithms, enabling the face template to deform well onto the defective face. Consequently, it can construct reconstructed nasal data that fit well with the surrounding soft tissues. In future research, the FMGen-Net model can be combined with the deformable template method, leveraging the ability of the artificial intelligence model to generate personalized faces and the deformable matching capability of the deformable template method. By using the personalized face generated by the FMGen-Net model as a template and assisting with the application of the deformable template method to deform it onto the defective face, the deformed facial data can be used as the 3D-TRF, thereby further improving the virtual reconstruction effectiveness of a personalized 3D-TRF for defects.

In the ‘morphological similarity’ evaluation of 20 cases of a full nasal-defect virtual reconstruction, we recorded the nasal subunits where the ‘max surface deviation’ was observed. Among these cases, the highest occurrences were in the nasal alae followed by the nasal tip, while the least frequent instances were noted in the nasal dorsum, nasal sidewalls, soft triangles, and nasal columella. This indicates that the proposed virtual reconstruction strategy for full nasal defects, centered around the FMGen-Net model, has room for improvement in the 3D anatomical restoration of nasal defects, particularly in nasal subunits with complex anatomical changes such as the nasal alae and nasal tip. 

In future research, more 3D facial data from different research centers can be employed to train the FMGen-Net model, enriching the artificial intelligence model’s learned prior knowledge of 3D facial shape features and enhancing the capability to construct personalized 3D-TRFs. Additionally, this study utilized an optimization algorithm to build a personalized 3D-TRF by using unidirectional chamfer distance as the loss function to calculate the difference between the generated facial point cloud data and the defective facial point cloud data, iteratively solving for the values of the latent space vector *z* to minimize the loss function and generate a reasonable face as the 3D-TRF. In future research, it is possible to explore other loss functions in the optimization algorithm, such as Earth Mover’s Distance (EMD). The accuracy of an EMD calculation is higher than that of chamfer distance [13], which may produce better optimization algorithm solution results.

In this study, we only conducted a preliminary assessment of the performance of the FMGen-Net model for the virtual reconstruction of full nasal defects. Due to FMGen-Net separating the tasks of learning 3D face generation and constructing a 3D-TRF for patients, it holds the potential to construct 3D-TRFs for any type of facial defect based on the learned prior facial-feature knowledge. In future work, additional testing of FMGen-Net in virtual reconstructions of other facial defects can be conducted to further validate the application of the FMGen-Net model in clinical settings.

## 5. Conclusions

This study introduced a novel method for the virtual reconstruction of full nasal defects. By training the deep learning model FMGen-Net and applying an optimization algorithm, we preliminarily achieved the automatic and personalized construction of a 3D-TRF. In the simulation experiment of 20 cases involving a full nasal defect reconstruction, the ‘3D surface deviation’ between the reconstructed nasal data and the original nasal data was 1.45 ± 0.24 mm. The ‘curve deviation’ of the reconstructed nasal data’s edge curve compared to the defective facial edge curve was 0.37 ± 0.09 mm. Our algorithm constructs nasal data that conform to the personalized anatomical morphology of the patient and fit well with the surrounding soft tissues. However, this study only conducted preliminary laboratory evaluations of our method. Further research is needed to assess its clinical effectiveness.

## Data Availability

The datasets used and analyzed during the current study are available from the corresponding author on reasonable request.

## Abbreviations

3D: three-dimensional; 3D-TRF: 3D target reference facial model
